# The Efficacy of Passive Ultrasonic Activation of Organic Solvents on Dissolving Two Root Canal Sealers

**DOI:** 10.22037/iej.2017.05

**Published:** 2017

**Authors:** Letícia Trevisan, Isadora Razzera Huerta, Carina Michelon, Mariana De Carlo Bello, Rafael Pillar, Carlos Alexandre Souza Bier

**Affiliations:** a *Department of Stomatology, Federal University of Santa Maria, Santa Maria, Rio Grande do Sul, Brazil; *; b *Graduate Program in Dental Science, Federal University of Santa Maria, Santa Maria, Rio Grande do Sul, Brazil*

**Keywords:** Eucalyptol, Orange Oil, Retreatment, Solvent, Ultrasound

## Abstract

**Introduction:**

The aim of this *in vitro *study was to evaluate the dissolving efficacy of eucalyptol and orange oil solvents associated with passive ultrasonic activation (PUA) in zinc oxide-eugenol (ZOE) based and epoxy resin-based root canal sealers.

**Methods and Materials:**

Seventy samples of each sealer were prepared and then randomized according to the solvent and the time of the ultrasonic activation (*n*=5). The mean amount of weight loss of sealers was calculated in percentages and was analyzed by using the Kruskal-Wallis and Bonferroni post-hoc tests.

**Results:**

The greatest values of weight loss were obtained with the ZOE sealer groups (*P*<0.05), regardless of the solvent that was used. An application of PUA for 3 min, with a renewal of orange oil solvent each min, showed the greatest percentage of weight loss in ZOE sealer compared to the others templates (*P*<0.05). Neither the solvent nor the different times had any influence on the weight loss of the resin sealer (*P*>0.05).

**Conclusion:**

The application of PUA with essential oils can be an effective method in dissolving ZOE based sealers.

## Introduction

The failure of endodontic therapy can occur especially by an improper cleaning of the root canal system and an inefficient hermetic sealing. Remnants of necrotic tissue or bacteria may remain in the root canal and be responsible for persistent periapical inflammation [1]. In these cases, endodontic retreatment is the primary choice to deal with residual or subsequently acquired infection [2].

The effective removal of the existing root filling material is essential during an endodontic retreatment in order to allow for an appropriate disinfection and adaptation of the new filling. Several techniques are already known for removing the filling material, including the use of solvents, heat producing appliances, hot hand instruments, mechanical methods and ultrasonic devices [-]. Nevertheless, it is also known that none of these methods completely remove the root filling material from the root canal [6, 7]. Passive ultrasonic activation (PUA) applied with different substances is being used as an auxiliary method in an attempt to improve the removal of gutta-percha and the sealers from the root canal systems [-]. It has been suggested that the term PUA is widely adopted in order to describe the actions that occur when a solution is agitated/activated by ultrasound energy without a simultaneous irrigation.

PUA potentiates the action of irrigants by means of ultrasonic waves that are generated from an instrument oscillating in the irrigating solution. This procedure improves the cleaning and disinfection of the root canal system and is similar to a passive ultrasonic irrigation, but without a simultaneous irrigation that uses a solution [11]. During retreatment, PUA can be used with organic solvents in an attempt to increase the dissolution of the root filling material and to improve the cleanliness of the root canal system [9]. However, the effectiveness of PUA that is associated with solvents when dissolving root canal sealers has never been evaluated.

There is a need for establishing a method that potentiates the dissolution of root canal sealers. As a result, the aim of this *in vitro* study was to evaluate the efficacy of PUA that is associated with eucalyptol and orange oil solvents on dissolving two endodontic sealers.

## Materials and Methods

Cylindrical acrylic molds with 8 mm diameter and 2 mm height were used to prepare 70 samples of each sealer. Two root canal sealers were prepared: an epoxy resin-based sealer (AH-Plus, Dentsply, DeTrey, Konstanz, Germany) and a zinc oxide-eugenol (ZOE) based sealer (Endofill, Dentsply Maillefer, Ballaigues, Switzerland). The molds were positioned on a glass plate by a double-sided adhesive tape in order to prevent an extravasation of the endodontic material. The inner wall of mold was lubricated with solid Vaseline petroleum jelly in order to facilitate the removal of the samples. The sealers were mixed according to the manufacturer’s instructions and were inserted into the acrylic molds. A polyester strip was placed on the upper surface of the sample to make sure that it was flat. This set was transferred to a chamber with an 80% relative humidity and a 37±1^°^C temperature for 72 h. The samples were then removed from the molds and the excess material was trimmed to the surface level of each sealer’s sample with a scalpel. The samples were weighed in grams (g) (up to four decimal places) on a precision scale (Coleman, Santo André, São Paulo, Brazil) that had been previously calibrated, in order to obtain their initial mass. The weights were recorded in triplicate and the mean values were calculated. The sealer samples were randomized for each solvent. Each solvent/sealer group was further divided into 7 equal subgroups (*n*=5) according to the PUA period of the solvents: 1 min/PUA; 2 min/PUA; 3 min/PUA; 4 min/PUA; 5 min/PUA; 3 min/REN/PUA (a 3-min activation with a renewal of the solvent at each min); 5 min/WO/PUA (a 5-min immersion of the sample in the solvent without PUA).

At room temperature, the samples were immersed in an adapted test tube with 1 mL of solvent, Eucalyptol (Biodinâmica, Ibiporã, Paraná, Brazil) or Orange Oil (Biodinâmica, Ibiporã, Paraná, Brazil). PUA was performed with a piezoelectric ultrasound (Ultra Sonic, Gnatus, Ribeirão Preto, São Paulo, Brazil) at full power and with an A90 adapter equipped with the instrument K-file #15 (Dentsply, Maillefer, Ballaigues, Switzerland). This procedure was conducted in order to avoid any contact of the endodontic instrument with the sample. In the 5 min/WO/PUA group, the samples were only immersed in the solvent for 5 min. Later, the samples were removed from the solvent and they were irrigated with 10 mL of distilled water. They were then blotted dry with an absorbent paper. The samples were kept in a dehumidifier for 24 h at 37±1^°^C and then they were weighed in triplicate.

The amount of lost sealer from each specimen was determined as the difference between the initial weight of the sealer and its final weight. The mean values and the standard deviations of the sealer dissolutions were calculated and were expressed in percentages for each solvent/sealer at each analyzed period of time. Statistical analyses were performed by using the STATA 12.0 software (StataCorp., CollegeStation, TX, USA) with the level of significance set at 0.05. The Shapiro-Wilk test showed a non-normal distribution of the data (*P*<0.05). Therefore, the weight loss of the sealers was analyzed by using the Kruskal Wallis test. The Bonferroni test was also performed as a post-hoc multiple comparison method.

## Results

The mean values and the standard deviations of a weight loss that were obtained for each solvent/sealer, at each analyzed time, with or without PUA, are presented in Table 1. 

The greatest values of a sealer weight loss were obtained by the ZOE sealer (*P*=0.00), regardless of the solvent that was used. The lowest values were obtained by the activation of orange oil with the resin sealer (*P*=0.00). 

When analyzing the different times in the orange oil/ZOE sealer group, PUA of the solvent for 3 min, with a renewal of the solvent at each min, showed the greatest percentages of the sealer’s weight loss that other times (*P*<0.05). In this same group, PUA for 1, 2 and 3 min presented the lowest values of weight loss and these were equivalent to the immersion of the samples in a solvent without an ultrasonic activation (*P*=1.00). 

In the eucalyptol/ZOE sealer group, PUA with renewal of the solvent was statistically superior only for PUA 1 min duration and for 5 min without PUA (*P*=0.01 and *P*=0.00, respectively).

In the orange oil/resin sealer group and in the eucalyptol/resin sealer group there were no statistical differences of a weight loss over the different time periods when they were analyzed (*P*=0.76 and *P*=0.19, respectively).

**Table 1.** The mean (SD) of weight loss inn each group

	**Orange oil**		**Eucalyptol**	
	**Endofill **	**AH-Plus**	**Endofill **	**AH-Plus**
**1 min/PUA**	2.20 (0.30)^A,a^	0.34 (0.35)^ A,b^	3.17 (1.65)^ AC,a^	1.55 (0.86)^ A,ab^
**2 min/PUA**	2.42 (0.46)^ A,a^	0.16 (0.06)^ A,b^	4.20 (0.89)^ ABC,c^	2.16 (1.33)^ A,a^
**3 min/PUA**	3.29 (0.41)^ AB,a^	0.27 (0.16)^ A,b^	5.16 (1.14)^ AB,c^	0.89 (0.96)^ A,b^
**4 min/PUA**	4.32 (1.10)^ BC,a^	0.40 (0.30)^ A,b^	3.87 (1.03)^ ABC,a^	0.81 (0.72)^ A,b^
**5 min/PUA**	5.01 (0.47)^ C,a^	0.30 (0.21)^ A,b^	4.21 (1.11)^ ABC,a^	0.64 (0.96)^ A,b^
**3 min/REN/PUA**	6.58 (1.29)^ D,a^	0.31 (0.12)^ A,b^	5.71 (0.44)^ B,a^	1.49 (0.81)^ A,b^
**5 min/REN/PUA**	2.39 (0.43)^ A,a^	0.27 (0.10)^ A,b^	2.21 (0.35)^ B,a^	1.06 (0.92)^ A,b^

*Uppercase* *letters: comparison of means among different time periods for the same sealer/solvent* *;* *-Similar letters indicates that there was no statistically significant differences (P>0.05); Lowercase letters: comparison of means among sealer/solvent for a specific time (row);* *-Similar letters indicates no statistically significant differences (P>0.05*

## Discussion

During an endodontic retreatment it is essential that the sealer be effectively removed from the entire root canal system [12]. The presence of endodontic sealers in the irregular spaces of the root canal, such as accessory canals, oval extensions, isthmii, and apical deltas, may hamper the cleaning and the disinfection, and hence, maintain microbial infections of the periapical tissues [13]. Therefore, this study aimed to evaluate the efficacy of PUA that is associated with eucalyptol and orange oil solvents on dissolving two endodontic sealers. Several techniques have been used to remove sealer and gutta-percha; however, none of these techniques can completely clean the root canal walls [14, 15], particularly in the apical third [16].

PUA activates and potentiates the effects of the solvent solution through the transmission of acoustic energy from an oscillating file. The energy is transmitted to the solution by means of ultrasonic waves that induce an acoustic streaming and cavitation [17]. This stimulates the creation, the expansion, and the contraction of bubbles in a liquid, intensifying the effect of the solution [18]. During a retreatment, the ultrasonic agitation of organic solvents can be beneficial for improving the chemical properties of these substances, and hence, they then increase their dissolving capacities for these root canal sealers [17]. 

The results of the present study have confirmed these facts. This research has shown that the association of an ultrasonic activation with organic solvents increases the actions of these solvents on the root canal sealers. According to Cavenago *et al.* [8]*, *the association of ultrasound with hypochlorite, after the use of the solvent during a retreatment, did not improve the removal of the filling material. Nevertheless, it should be emphasized that these authors only activated the sodium hypochlorite.

Through ultrasound activation, it can be expected that the irrigant penetrates more easily into those areas that are difficult to clean, as the apical is a third of the root canal system [19], and thus, it increases the effectiveness of the disinfection [20]. However, the association of ultrasound with a solvent can be questioned by the possibility of promoting an extrusion of the solvent and causing deleterious effects on the periapical tissues. Nevertheless, it has been demonstrated that PUA does not increase the apical extrusion [21].

Some solvents, such as xylene or chloroform, are very effective in the dissolution of sealers [5, 22]. However, they are associated with a high toxicity to the periapical tissues [23, 24]. When trying to minimize this conflict between the effectiveness and the toxicity, essential oils have been used as solvents without harmful effects, regardless of their concentrations [3, 5, 25], justifying their choice in this study.

The results of the present study have indicated that PUA with orange oil for 3 min, with renewal of the solvent at each min, was more effective when dissolving the ZOE sealer than other times. This probability can be explained by the renewal of the solvent, which avoided its saturation, and as a result, it promoted higher sealer dissolutions. Besides, the activation of ultrasound caused an elevation in the temperature of the solvent (2^°^C) [26, 27] and that probably improved its ability to dissolve the ZOE sealer group, since its effectiveness rises at a higher temperature [23, 28].

Regarding the effects of PUA with eucalyptol in the dissolution of the ZOE sealer, the weight loss of the sealer was higher when the PUA was used for 3 min with a replacement of the solvent. However, there was a statistically significant difference with the group that did not use the PUA when compared with the group in which the PUA was only used for 1 continuous min. Therefore, the root canals that were filled with the ZOE sealer and when they used eucalyptol during the retreatment, a procedure must be suggested to use the PUA solvent for more than 1 min, in order to enhance the dissolution of the sealer.

When considering the resin sealer, regardless of the solvent used, no statistically significant difference was perceived at the analyzed times. In other words, using or not using PUA, regardless of renewing the solvent, did not improve the dissolution of the resin sealer. This result can be explained by the sealer’s composition. Some authors have stated that this cement has an extremely low dissolution capacity when using organic solvents [4, 29]. Furthermore, resin sealers present good mechanical properties and a cohesive resistance, which makes them more difficult to detach the endodontic sealer from the dentin wall when using PUA [30]. 

The composition of the sealers can also explain the higher dissolution of the ZOE sealer than the resin sealer. Endofill is a ZOE-based sealer, which has a higher solubility when compared to the resin sealer [3]. Poggio *et al*. [31] verified that the ZOE sealer presented solubility near the desired maximum limit (3% mass fraction).

The times of the ultrasonic activation that were used in this present study were determined by taking into consideration the clinical practices of a dentist. A period longer than 5 min would be non-viable for the performance of this protocol, as it would make the appointment overly long. Therefore, the maximum period of time that was established in this study was 5 min. The renewal of the solvent was set at 3 min, as it was an intermediate and clinically viable period. 

This *in vitro *study has presented limitations, such as the lack of a chemical analysis of the solvents. This would be in order to quantitatively verify the saturation of the solutions and the interaction pattern of root canal sealers with solvents during PUA. Thus, further research should be conducted in order to try to solve these gaps. 

## Conclusion

In conclusion, the main results of this study demonstrate that PUA with essential oils can effectively dissolve ZOE sealer when renewal of the solvent is administered. 
